# OMRT-12. Nanoparticle-based CRISPR-Cas9 delivery for anti-glioblastoma immunotherapy

**DOI:** 10.1093/noajnl/vdab070.036

**Published:** 2021-07-05

**Authors:** Rocio Aguilar, Javier Fierro, Joshua Perez, Huanyu Dou

**Affiliations:** Texas Tech University Health Sciences Center El Paso, El Paso, TX, USA

## Abstract

Anti-glioblastoma GBM) immunotherapy poses a great challenge due to immunosuppressive brain tumor environments and the blood brain barrier (BBB). Programmed death ligand 1 (PD-L1) is an immune checkpoint that mediated the immune resistance. Inhibition of PD-L1 by antibodies was widely studied to treat many type of cancers. However, the inefficient therapeutic immune response became a significant barrier for treatment of GBM. CRISPR/Cas9 gene editing can be used to knockout both membrane and cytoplasmic PD-L1, leading to an enhanced immunotherapeutic strategy. It is extremely difficulty to deliver CRISPR/Cas9 containing plasmid for translational and clinic applications. We have been developed a core-shell nanoparticle (NP) to carry CRISPR/Cas9 plasmid for PD-L1 knockout. The different NP formulations were made and optimized to deliver CRISPR/Cas9 plasmid. NPs were prepared by modifying the water temperature, sonication power and time and formulation time. The obtained NPs had a size of 115-160nm and a charge of 40-50mV. The size and charge were significantly altered after CRISPR/Cas9 plasmids were loaded into NPs (Cas9-NPs). Agarose gel electrophoresis showed that CRISPR/Cas9 plasmids were fully encapsulated by NPs with 1 and 2 ug. The positive DNA bands occurred with 4ng, indicating the overloaded CRISPR/Cas9 plasmid. Fluorescence microscopy determined Cas9-NPs uptake by U87 cells under a time-dependent manner. GFP tagged Cas9-NPs were treated to U87 cells for transfection evaluation. The obtained different NPs delivery of CRISPR/Cas9 exhibited various transfection efficiencies in U87 cells. Visualization of intracellular Cas9-NPs showed increases in uptake by U87 cells from 0.5, 1, 2, and 4 hours. The greater nuclear accumulation of Cas9-NPs was seen at 24 hours. A western blot assay determined the success of PD-L1 deletion by Cas9-NPs in human GBM U87 cells. NPs-based CRISPR/Cas9 gene-editing system has great potential as an immunotherapeutic platform to treat GBM.

